# Fourier Transform Infrared (FTIR) Spectroscopic Analyses of Microbiological Samples and Biogenic Selenium Nanoparticles of Microbial Origin: Sample Preparation Effects

**DOI:** 10.3390/molecules26041146

**Published:** 2021-02-21

**Authors:** Alexander A. Kamnev, Yulia A. Dyatlova, Odissey A. Kenzhegulov, Anastasiya A. Vladimirova, Polina V. Mamchenkova, Anna V. Tugarova

**Affiliations:** Laboratory of Biochemistry, Institute of Biochemistry and Physiology of Plants and Microorganisms, Russian Academy of Sciences, 410049 Saratov, Russia; jdyatlowa2013@yandex.ru (Y.A.D.); odissey94.sid@mail.ru (O.A.K.); vladimirova-nastyusha@bk.ru (A.A.V.); norgeadress@gmail.com (P.V.M.); tugarova_anna@mail.ru (A.V.T.)

**Keywords:** sample preparation, FTIR spectroscopy, bacterial biomass, biogenic selenium nanoparticles, *Azospirillum brasilense*, *Azospirillum baldaniorum*

## Abstract

To demonstrate the importance of sample preparation used in Fourier transform infrared (FTIR) spectroscopy of microbiological materials, bacterial biomass samples with and without grinding and after different drying periods (1.5–23 h at 45 °C), as well as biogenic selenium nanoparticles (SeNPs; without washing and after one to three washing steps) were comparatively studied by transmission FTIR spectroscopy. For preparing bacterial biomass samples, *Azospirillum brasilense* Sp7 and *A. baldaniorum* Sp245 (earlier known as *A. brasilense* Sp245) were used. The SeNPs were obtained using *A. brasilense* Sp7 incubated with selenite. Grinding of the biomass samples was shown to result in slight downshifting of the bands related to cellular poly-3-hydroxybutyrate (PHB) present in the samples in small amounts (under ~10%), reflecting its partial crystallisation. Drying for 23 h was shown to give more reproducible FTIR spectra of bacterial samples. SeNPs were shown to contain capping layers of proteins, polysaccharides and lipids. The as-prepared SeNPs contained significant amounts of carboxylated components in their bioorganic capping, which appeared to be weakly bound and were largely removed after washing. Spectroscopic characteristics and changes induced by various sample preparation steps are discussed with regard to optimising sample treatment procedures for FTIR spectroscopic analyses of microbiological specimens.

## 1. Introduction

The Fourier transform infrared (FTIR) spectroscopic technique is versatile and sensitive to the molecular composition and fine structural features, as well as intra- and intermolecular interactions, of functional groups in samples virtually in all aggregation states. This has made it indispensable for both theoretical and experimental structural and spectrochemical analytical studies of diverse materials ranging from small molecules (see, e.g., [1,2,3,4,5,6]) to more complicated materials, macromolecules and supramolecular structures [7,8,9,10,11], up to prokaryotic or eukaryotic cells and tissues [12,13,14,15,16,17,18]. Over recent decades, FTIR spectroscopy has been increasingly used in microbiological studies for the identification and classification of microorganisms, as well as for solving various bioanalytical problems related to microbiology [12,13,15,19,20,21,22,23,24,25]. Nevertheless, standardised sample preparation for biological objects, including microbial cells, as well as mathematical methods for analysing the resulting complicated spectra, are still under development. To date, a few topical articles have been published in which the preparation of microbiological samples for analysis by using FTIR spectroscopy and some specific features of the technique are discussed (see, e.g., [19,20,22,23,24]). Nevertheless, further development and optimisation of methodologies for preparing various microbiological samples for FTIR spectroscopic analysis are still of significance to ensure obtaining reliable spectroscopic data. The latter are indispensable for the most meaningful interpretation adequately reflecting the objects under study.

In this report, we consider some methodological approaches in sample preparation and their effects when using FTIR spectroscopy as applied to bacterial cultures (dried biomass) and biogenic selenium (Se^0^) nanoparticles (SeNPs) of bacterial origin. In our work, two widely studied strains were used which belong to the genus *Azospirillum*, Gram-negative alphaproteobacteria, among which there are many ubiquitous rhizobacteria with phytostimulating capabilities and a number of other biotechnologically attractive traits (for reviews, see, e.g., [26,27,28], as well as some of our earlier experimental reports [21,24,25] and references cited therein). FTIR spectroscopy in its various variants is a useful technique providing a wealth of information on their ecology and physiological behaviour, particularly under stress conditions [21,24,25]. The strains under study in this work, *A. brasilense* Sp7 [29] and *A. baldaniorum* Sp245 (earlier known as *A. brasilense* Sp245 [30] and reclassified only recently [31]), have also been documented to be capable of reducing selenite (Se^IV^O_3_^2−^) with the formation of SeNPs [32,33]. This trait, which is common for a number of microorganisms [34,35], is of importance for agrobiotechnology (e.g., bioremediation of seleniferous soils and aquifers) and nanobiotechnology (green synthesis of biogenic SeNPs and other Se-containing nanostructures) [35]. Thus, analysing such biogenic SeNPs using instrumental techniques, including FTIR spectroscopy, particularly with regard to the bioorganic capping layer of such nanostructures [34,36], is of primary importance.

## 2. Results and Discussion

### 2.1. Bacterial Biomass: Sample Treatment Effects in FTIR Spectroscopic Analysis

To date, large amounts of results and data obtained using FTIR spectroscopy have made it possible to form an extensive database for the analysis and interpretation of FTIR spectra of various microbiological objects. Nevertheless, as noted above, many aspects of sample preparation of such samples, which can commonly be structurally and compositionally complicated and non-uniform, are still not fully standardised. As has been mentioned earlier (see, e.g., [19,20,24,25] and references therein), FTIR spectra of microbial biomass can be obtained in various ways. Each way of sample preparation has its peculiarities which, if even slightly altered, may result in some changes (sometimes directly visible or resolvable using special approaches) in spectroscopic images, reflecting some structural changes in the sample. Therefore, it is of importance to have information on how various sample preparation steps can influence the resulting spectra and ultimately to standardise and develop a valid methodology for preparing bacterial samples for a reliable FTIR spectroscopic analysis. In this work, our attention was directed to some specific and important processing steps (grinding and drying) and to studying their effects on the measured FTIR spectra in the case of bacterial samples.

#### 2.1.1. Effects of Grinding

Prior to measurement, the dried bacterial culture was pretreated in two variants: (1) the sample was thoroughly powdered (ground in a mortar), and the resulting powder was resuspended in Milli-Q water and processed as described in Section 3.3; (2) the grinding stage was excluded from sample preparation, so that the dry biomass was directly processed as described in Section 3.3. Traditionally, the grinding stage is an important part of sample preparation in the FTIR spectroscopy of materials (especially non-uniform or heterogeneous materials), since it allows for obtaining a more homogeneous aqueous or oil suspension. In the case of an aqueous suspension (as used by us previously [25] and in this work), when dried, it forms a uniform thin film on ZnSe glasses. Such films make it possible to obtain high-quality transmission FTIR spectra with a high signal-to-noise ratio and, therefore, to greatly facilitate further analysis of the data obtained. (Note that, while grinding, part of material may be lost, which has to be taken into account when the amounts of samples are limited.)

**Figure 1:**
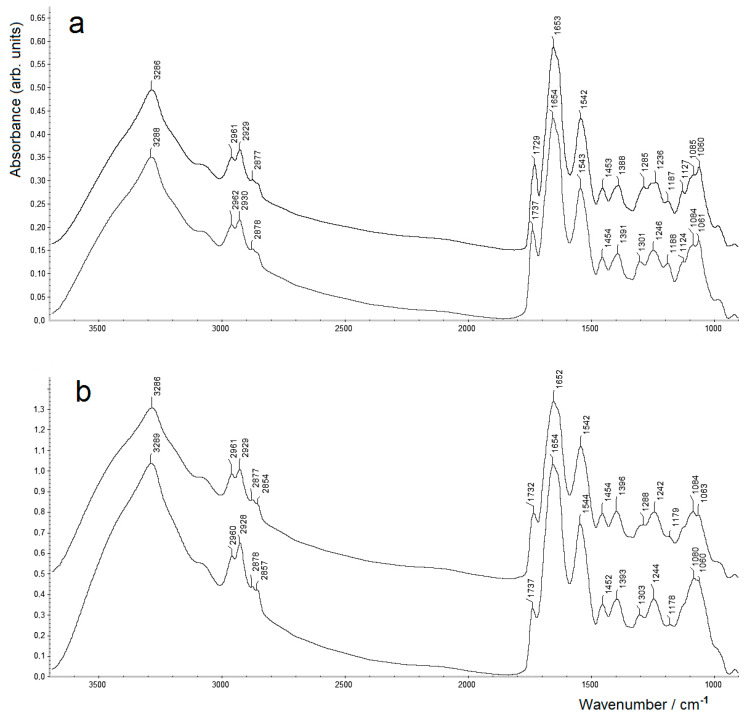
Transmission FTIR spectra of dry bacterial biomass of *Azospirillum baldaniorum* Sp245 (**a**) and *A. brasilense* Sp7 (**b**) measured with grinding (upper spectra) or without grinding (lower spectra). The upper spectra are vertically shifted from the baseline (zero absorbance) for clarity.

Figure 1 shows FTIR spectra of *A. baldaniorum* Sp245 and *A. brasilense* Sp7 biomass samples (dried for 23 h) with and without grinding. As can be seen, the spectra contain all the bands typical of bacterial FTIR spectra [19,20] (see Table 1 for band assignments of typical bands for *A. baldaniorum* Sp245), and all the spectra generally look very similar.

In the FTIR spectra of the samples in Figure 1, some differences (exceeding the spectral resolution of 4 cm^−1^) are observed only in the region around ~1730 cm^−1^, as well as ~1300 cm^−1^. For the samples subjected to grinding, the values of the maxima of these bands are noticeably lower. Thus, for *A. baldaniorum* Sp245, the bands observed at 1737 and 1301 cm^−1^ shifted to 1729 and 1285 cm^−1^, respectively, after grinding (see Figure 1a). A similar shift is observed for *A. brasilense* Sp7 (the bands observed at 1737 and 1303 cm^−1^ shifted to 1732 and 1288 cm^−1^, respectively; see Figure 1b).

It is common knowledge that the aforementioned bands correspond to the functional groups of the intracellular reserve biopolyesters of the polyhydroxyalkanoate (PHA) series [19,37], which in azospirilla are represented by the homopolymer poly-3-hydroxybutyrate (PHB) (see [21,24,25] and references cited therein). In PHB, polyester chains are interconnected by weak C–H···O hydrogen bonds [37]. Changes in the intensity of inter- and intramolecular interactions formed by these hydrogen bonds between the ester carbonyl group (showing a band at ca. 1720–1750 cm^−1^ due to C=O stretching vibrations, which are sensitive to H-bonding) and the –CH_3_ group in the polymer chains cause some variability in the degree of ordering (crystallinity), which is one of the most important properties of native PHB. As the degree of ordering decreases, the aforementioned bands in FTIR spectra shift to higher frequencies, and vice versa [37,38,39]. (In our case, a shift is also observed in the region of ~1240 cm^−1^ related in part to C–O–C vibrations of ester moieties.)

It has to be mentioned that the capability of PHA biosynthesis and accumulation as intracellular granules is of primary importance for bacterial survival and stress endurance [40,41,42,43,44]. Bacteria of the genus *Azospirillum* are known to be capable of accumulating relatively large amounts of PHB which, under appropriate conditions (e.g., lack of bound nitrogen, i.e., a high C:N ratio in the medium), may exceed 60–70% of dry cell weight (d.c.w.) [21,25,45]. Thus, as follows from the spectra (see Figure 1), the relative amounts of intracellular PHB in the samples studied in this work are low, around or below ~10% d.c.w. (cf., e.g., [25]), because of the presence of a minimal normal concentration [21] of bound nitrogen (as NH_4_^+^) in the culture medium (see Section 3.1). Nevertheless, the observed downshifting of several PHB-related bands in samples after grinding (vide supra) to slightly but statistically significantly lower wavenumbers unambiguously show that the grinding step induces partial transition from the metastable and more amorphous state of the intracellular PHB to its more ordered state (i.e., its partial crystallisation). Note that a very similar but even more strongly expressed downshifting of the main PHB-related bands, ν(C=O) around ~1740 cm^−1^ and ν(C–O–C/C–C–O) at ~1300 cm^−1^, was shown to be induced by the sample preparation procedure that involves grinding and pressing the bacterial biomass with KBr [24] often used in FTIR spectroscopy.

#### 2.1.2. Effects of Drying

Another variation in sample preparation conditions tested in this work was associated with the duration of drying. The presence of water (which is featured by strong vibration bands, particularly the “scissoring” mode of δ(H–O–H) vibrations at ~1640–1650 cm^−1^ which falls within the amide I region of proteins [19,46]), even in traces, in a sample can alter the measured FTIR spectrum.

**Figure 2:**
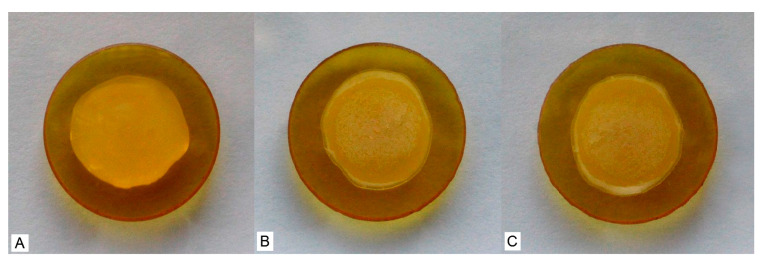
Photographs of aqueous suspension of *A. brasilense* Sp7 bacterial biomass freshly applied to a ZnSe glass (**A**), dried for 1.5 h (**B**) and for 23 h (**C**).

The drying periods for the bacterial samples adopted in this study were 1.5 h and 23 h (at 45 °C). Photographs of the samples just applied to the ZnSe glass and dried for 1.5 h and 23 h are shown in Figure 2.

**Figure 3:**
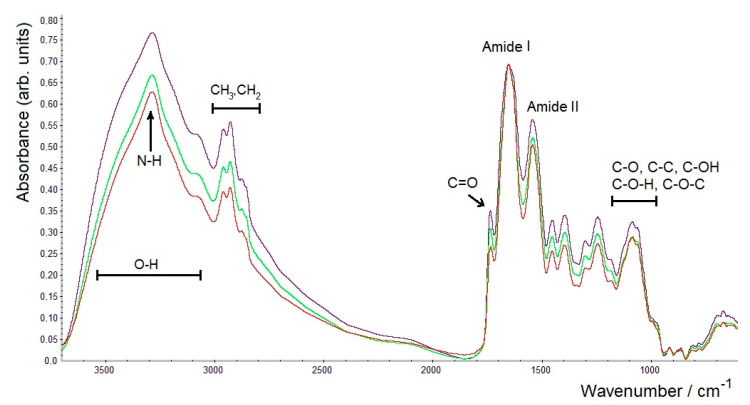
FTIR spectra of three samples of *A. brasilense* Sp7 biomass dried for 1.5 h (3 replicates) normalised by the amide I band.

From the same sample of the dried bacterial culture of *A. brasilense* Sp7, three separate ZnSe glasses were prepared for FTIR spectroscopy of these parallel measurements. The amide I band (~1645–1655 cm^−1^, peptide bonds in proteins) was used as a normalisation standard for the FTIR spectra. As can be seen from Figure 3, some differences were observed in the intensities for the three parallel samples (each dried for 1.5 h) in the region of the broad band at 3700–2700 cm^−1^ (the region of stretching vibrations of O–H and N–H groups), as well as in the region of 1485–1000 cm^−1^ (C–O, C–C, C–O–H, C–O–C in polysaccharides and polyesters).

**Figure 4:**
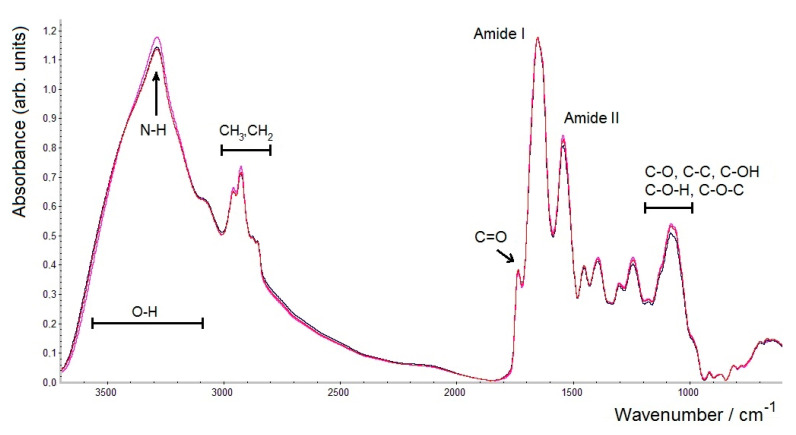
FTIR spectra of three samples of *A. brasilense* Sp7 biomass dried for 23 h (3 replicates) normalised by the amide I band.

However, the intensities of all the bands for three similar replicate samples of *A. brasilense* Sp7 biomass dried for 23 h were virtually the same (Figure 4). It is important to emphasise that, despite some differences in the intensities of the bands in the FTIR spectra between shorter (1.5 h) and longer (23 h) drying periods, the positions of the maxima of all bands in the FTIR spectra of all samples remained unchanged. Thus, with a longer drying time (23 h in our case), greater reproducibility was observed in measuring transmission FTIR spectra with regard to absorption band intensities. Accordingly, it may be recommended to dry bacterial samples being prepared for FTIR spectroscopic analysis at moderate temperatures up to 40–45 °C (to avoid denaturation of proteins) overnight to ensure a good reproducibility of both the intensities and band positions.

It is also worth noting a specific difficulty that we encountered in our work. In some cases, sample preparation of the bacterial biomass for transmission FTIR spectroscopic measurements (for obtaining aqueous suspensions to be applied to a ZnSe glass) may be hampered both in the case of grinding and without it. This is observed when bacteria have accumulated significant amounts of PHB (e.g., over ~40% d.c.w.). Since this polyester has hydrophobic properties, and its physical properties are similar to those of some commercial plastics, large PHB amounts in bacterial culture lead to difficulties in the process of sample preparation; the culture becomes difficult to grind and is also practically not resuspendable in Milli-Q water [25]. Such difficulties in sample preparation require a special approach to bacterial biomass with a high PHB content and the development of additional steps that would make it possible to obtain the most homogeneous sample appropriate for FTIR measurements.

Thus, in this part, it has been shown that varying the conditions in some stages of sample processing, such as drying time, as well as the use of the grinding of bacterial biomass samples, can lead to changes in the obtained FTIR spectra when analysing microbiological objects. Consequently, the application and details of such steps can be optimised with regard to the expected composition of the bacterial specimens, which has been attempted in this study.

### 2.2. Analysis of Bacterially Synthesised Selenium Nanoparticles by FTIR Spectroscopy

In this part of the work, using the example of biogenic SeNPs, we discuss the influence of the sample preparation process on the state of the samples reflected in their FTIR spectra. The isolated SeNPs of bacterial origin obtained using *A. brasilense* Sp7, with different numbers of washing steps, were studied by FTIR spectroscopy.

**Figure 5:**
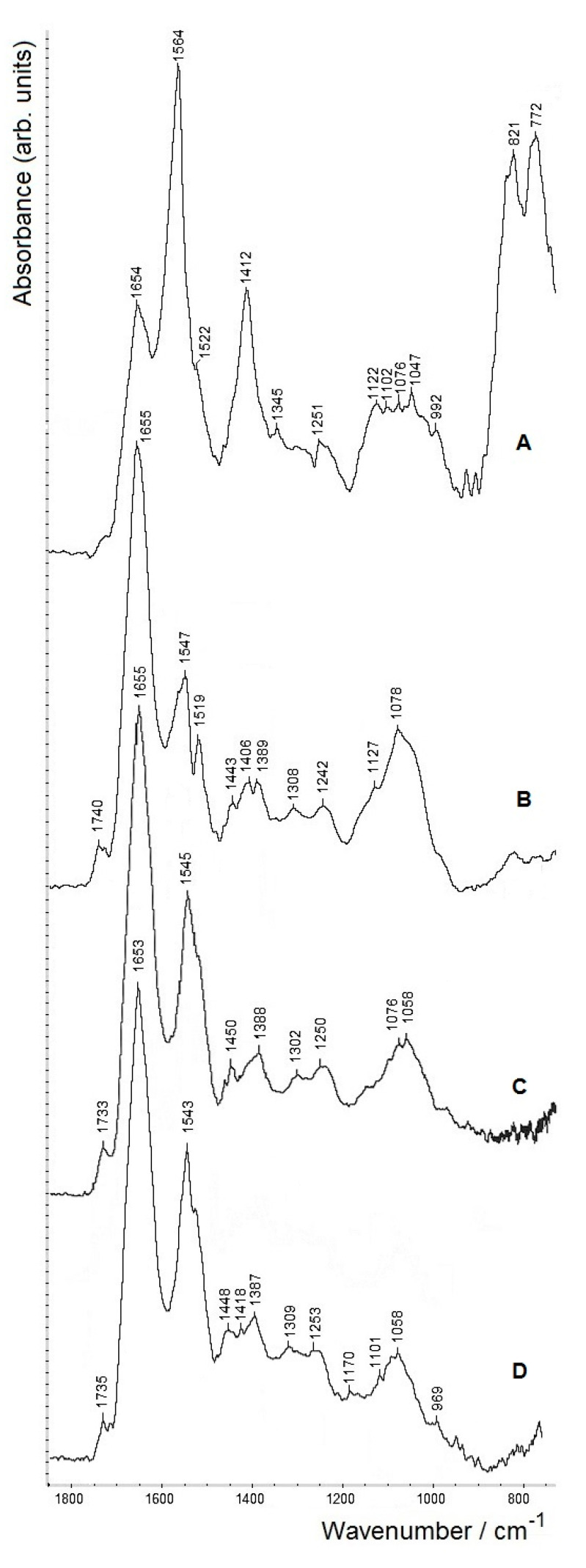
FTIR spectra (transmission mode, on ZnSe discs) of isolated biogenic selenium nanoparticles (SeNPs) obtained using *A. brasilense* Sp7, measured without washing (**A**) and after 1 (**B**), 2 (**C**) and 3 (**D**) washing steps. Spectra **A**–**C** are vertically shifted from the baseline (which corresponds to spectrum **D**) for clarity.

Figure 5 shows the region 2000–700 cm^−1^, which reflects the greatest changes in the samples under study and is most informative in FTIR spectra when studying biological samples. Note that the FTIR spectra of samples with two and three washing steps, owing to a partial loss of material occurring during the purification of SeNPs performed by washing, are characterised by a lower signal-to-noise ratio, which has led to some increase in noise in the FTIR spectra, as can be seen in spectra C and D under 1000 cm^−1^ (however, not impairing the analysis).

First of all, from Figure 5, it is clearly seen that the FTIR spectrum of isolated biogenic SeNPs without additional washing steps (spectrum **A**) is noticeably different from FTIR spectra of those after one to three washing steps (see spectra **B**–**D** which have much fewer differences between them). As has been well documented, microbially synthesised SeNPs always contain specific capping layers of biomacromolecules originating from the biological system in which they were synthesised [34,35,36]. We performed a comparative analysis of the spectra in Figure 5 (as the SeNPs were obtained using *A. brasilense* Sp7, they are expected to contain bioorganic components from this bacterium; hence part of the assignments listed in Table 1, which are typical of bacterial cell biomass, may be used).

For the FTIR spectrum of nanoparticles that were not washed (spectrum **A**), as compared to the other spectra, the most significant difference is the presence of a strong band at 1564 cm^−1^. This band may be assigned to antisymmetric stretching vibrations of ionised carboxylate residues (salts of carboxylic acids), ν_as_(COO^−^), in the biomacromolecular shell of SeNPs. This assignment is also confirmed by the presence of the accompanying band related to the symmetric stretching vibrations ν_s_(COO^−^) at 1412 cm^−1^, as well as of the bands related to its bending vibrations δ(COO^−^) (at 821 and 772 cm^−1^). The carboxylates may evidently represent various amino acid residues and be contained in carboxylated polysaccharides (the typical polysaccharide region within 1200–950 cm^−1^ is also seen in spectrum **A**). Note that the positions of carboxylate-related vibration bands are known to vary depending on the interactions with the surrounding biomolecules. The band at 1654 cm^−1^ in spectrum **A** represents the amide I region of proteins (see below); the accompanying amide II band around 1540 cm^−1^ is definitely overlapped by the strong and broad ν_as_(COO^−^) absorption. Very similar results were reported earlier for biogenic SeNPs isolated from *A. brasilense* Sp7 biomass without the additional washing steps [47].

As can be seen in spectrum **B**, the amount of carboxylic residues significantly decreased after the first washing step; further washing brings about only minor changes (spectra **C**,**D**). Thus, the carboxylate-containing components are most likely rather weakly bound to the surface of SeNPs, in contrast to the rest of the biomacromolecular shell, which evidently remains stable. This is in line with the recently reported comparative data on SeNPs of bacterial origin, where the bioorganic capping layers (showing differences when obtained using different bacteria) are postulated to contain an outer, more weakly bound shell and an inner part more strongly bound to the Se core [36].

In spectra **B**–**D**, both amide I (1655–1653 cm^−1^) and amide II (1547–1543 cm^−1^) bands related to proteins are more pronounced. The polysaccharide region (1200–950 cm^−1^) is slightly diminishing with each additional washing step (cf. spectra **B**–**D**), indicating that part of carboxylic groups may indeed be associated with weakly bound carboxypolysaccharides removed upon washing. Besides proteins and polysaccharides, the presence of lipids at all steps is corroborated by the typical ester ν(C=O) band around 1740 cm^−1^ (which in spectrum **A** is seen as a weaker shoulder and appears to be somewhat more pronounced after even the first washing; cf. spectra **B**–**D**).

**Figure 6:**
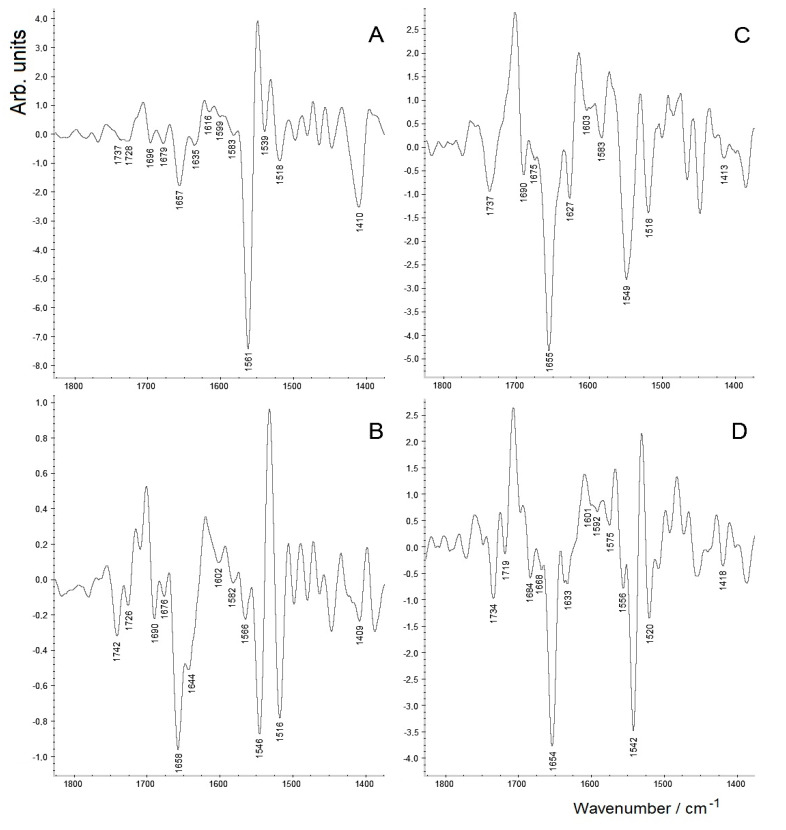
Second derivatives of FTIR spectra of isolated biogenic SeNPs obtained using *A. brasilense* Sp7 (see Figure 5) measured without washing (**A**) and after 1 (**B**), 2 (**C**) and 3 (**D**) washing steps.

In order to reveal unresolved (closely overlapping) bands, second derivatives of the spectra can be informative, especially for complicated spectra of microbiological samples [19,48]. Using OMNIC software, the second derivatives of the FTIR spectra shown in Figure 5 were calculated and presented in the most informative spectroscopic region (~1800–1400 cm^−1^; Figure 6, Table 2). Minima on the second derivatives (below zero point) correspond to both well-resolved spectral bands and inflection points (poorly resolved bands that may be seen as shoulders, overlapping with stronger adjacent bands) in the original spectrum [19].

As can be seen from Figure 6, the two typical protein bands, amide I and amide II, in curve **A** (SeNPs separated without additional washing) at 1657 and 1539 cm^−1^, respectively, are weaker than the two dominating peaks assigned to ν_as_ and ν_s_ of carboxylate (at 1561 and 1410 cm^−1^). The ester ν(C=O) band in curve **A** (split into two components, at 1737 and 1728 cm^−1^) is also very weak. However, after the first washing, when a significant part of the carboxylate-containing components have evidently been removed, and after the next two to three washing steps, the protein bands at ~1650 and ~1540 cm^−1^, as well as the ν(C=O) bands related to lipids (at ~1740 cm^−1^), are much more clearly seen (see curves **B**–**D**). It is necessary to add that, owing to the presence of carboxylic groups, especially in the form of ionised carboxylates (salts which dissociate in solution), in the surface capping layer of biogenic SeNPs, the latter are most often characterised by negative zeta potentials which stabilise their aqueous suspensions [33,34,36].

It may also be noted that for the amide I band, which is sensitive to the secondary structure of protein and is known to contain several closely overlapping bands within the region ~1690–1620 cm^−1^ [19,21], besides the main band within 1658–1654 cm^−1^ (the region typical of the dominating α-helix), there are several weaker component bands within the “full” amide I region clearly seen in curves **B**–**D** in Figure 6, which correspond to several β-structured protein components [19,21,48]. (Their detailed discussion is, however, out of the scope of this paper.)

Thus, it has been shown that, in the case of biogenic SeNPs, their sample preparation for FTIR spectroscopic analysis is an important step. As has been found, additional washing (even one step) decreases the content of weakly bound carboxylic components in the sample, which is reflected in the FTIR spectra. On the one hand, generally during sample preparation, it is necessary to take into account that the components of the buffer or medium can contribute to the measured spectrum. On the other hand, the procedures for removing these components, particularly the widely used method of washing a biological sample in combination with centrifugation, can concomitantly lead to a change in its state and/or composition, which is reflected in FTIR spectra, as shown in this work.

## 3. Materials and Methods

### 3.1. Bacterial Strains and Growth Conditions

Wild-type strains *Azospirillum brasilense* Sp7 [29] (ATCC 29145) and *Azospirillum baldaniorum* Sp245 [31] (previously known as *Azospirillum brasilense* Sp245 [30]) were taken from the Collection of Rhizosphere Microorganisms [WDCM 1021] maintained at the Institute of Biochemistry and Physiology of Plants and Microorganisms, Russian Academy of Sciences, Saratov, Russia [49] (URL: http://collection.ibppm.ru/catalogue/azospirillum/azospirillum-brasilense/ accessed on 13 January 2021). The bacteria were cultivated in a liquid modified malate salt medium (MSM) as reported earlier [24,25] which contained the following salts (g∙L^–1^): K_2_HPO_4_, 3.0; KH_2_PO_4_, 2.0; NH_4_Cl, 0.5; NaCl, 0.1; FeSO_4_∙7H_2_O, 0.02 (added as chelate with nitrilotriacetic acid); CaCl_2_, 0.02; MgSO_4_∙7H_2_O, 0.2; Na_2_MoO_4_∙2H_2_O, 0.002; sodium malate, 5.0 (obtained by mixing 3.76 g of malic acid with 2.24 g NaOH per litre), yeast extract, 0.1, pH 6.8–7.0. The cultures (100 mL in 250 mL Erlenmeyer flasks) were grown under aerobic conditions on a shaker (180 rpm) for up to 19 h. Cell growth was monitored at λ = 595 nm (Spekol 221, Germany); the optical density (A_595_) values of the resulting culture suspensions were about 1.0.

### 3.2. Bacterial Synthesis of SeNPs and Their Purification

The SeNPs were obtained according to the procedure reported elsewhere [47] with minor modifications. Briefly, bacterial cells of *A. brasilense* Sp7 (grown as described in Section 3.1) were harvested by centrifugation in 2 mL Eppendorf tubes (Minispin centrifuge; 15 min, 7000*g* ×) and washed three times with sterile saline solution (0.85% NaCl aqueous solution) to remove the culture medium components. All the next steps were performed under sterile conditions. The resulting wet biomass pellet was resuspended in half of the initial volume of sterile saline solution. Sodium selenite (Na_2_SeO_3_∙5H_2_O, “Merck”) as 0.5 M stock aqueous solution was added to the suspensions up to 10 mM. Suspensions containing the cells (washed as above) and selenite were placed in a thermostat (at 31–32 °C). The SeNPs thereby formed were monitored by transmission electron microscopy (TEM; data not shown; see, e.g., [33,47]). After 24 h, the bacterial cells were removed from the suspension by “soft” centrifugation (1400*g* ×, 5 min); the supernatant with SeNPs was collected and filtered through a 0.22 or 0.44 mm PVDF filter to remove occasional bacterial cells. The suspensions of SeNPs were further centrifuged at 12,000*g* × for 30 min, and the collected precipitate pellet was resuspended in a minimum volume of Milli-Q directly for FTIR spectroscopic analysis (on a ZnSe disc) or after 1 to 3 additional washing steps in Milli-Q water and centrifugation at 12,000*g* × for 30 min.

### 3.3. Sample Preparation for FTIR Spectroscopic Analyses

The bacterial cells of *A. brasilense* Sp7 and *A. baldaniorum* Sp245 (see Section 3.1) were collected by centrifugation (10,000*g* ×, 10 min, 4 °C), washed 3 times with physiological solution and dried (on open Petri dishes, ø 3.5 cm) in a thermostatted desiccator at 45 °C up to a constant weight. For infrared spectroscopic measurements, the samples of dried biomass were prepared in several ways: with/without grinding and with different drying periods (1.5 or 23 h). As for grinding, dry bacterial biomass was powdered in a small agate mortar (for about 5 min). The bacterial samples were resuspended in a small volume of Milli-Q water. SeNPs were prepared as described above (Section 3.2). Then the resulting aqueous suspensions (about 30–70 μL) were placed as thin films on clean flat ZnSe discs (CVD-ZnSe, “R’AIN Optics”, Dzerzhinsk, Russia; ø 1.0 cm, thickness 0.2 cm) and dried at 45 °C again as described above.

### 3.4. FTIR Spectroscopic Measurements

Transmission FTIR spectroscopic measurements were performed as described elsewhere [47] on a Nicolet 6700 FTIR spectrometer (Thermo Electron Corporation, Waltham, MA, USA; DTGS detector; KBr beam splitter). Spectra were collected with a total of 64 scans (resolution 2 cm^–1^ for spectra of SeNPs and 4 cm^−1^ for bacterial biomass samples) against the ZnSe disc background and manipulated using OMNIC software (version 8.2.0.387, Thermo Electron Corporation, Waltham, MA, USA) supplied by the manufacturer of the spectrometer. For each spectrum, the baseline was corrected using the “automatic baseline correct” function, and then each spectrum was smoothed using the standard “automatic smooth” function of the software which uses the Savitsky–Golay algorithm (95-point moving second-degree polynomial). All the FTIR spectroscopic measurements were repeated two (for SeNPs) or three times (for bacteria) for each sample and were well reproducible.

## 4. Conclusions

It has been shown that preliminary sample preparation steps (such as grinding, washing and drying) of microbiological specimens for transmission FTIR spectroscopic measurements may in some cases alter the composition and other properties of samples, which is reflected in their FTIR spectra. Thus, special care should be taken to ensure that samples are analysed by FTIR spectroscopy in their stable state which would ensure obtaining reproducible spectra. However, if any sample preparation step is expected to alter the properties of the samples under study, this can be checked by comparing their FTIR spectra before and after a sample treatment step. For complicated microbiological objects (such as bacterial biomass or nanoparticles of microbial origin, as studied in this work), the described examples could allow a most adequate protocol for sample preparation to be chosen to ensure obtaining reliable and reproducible spectroscopic data.

## Figures and Tables

**Table 1:** Band maxima of typical vibration bands in FTIR spectra of dry biomass samples of *A. baldaniorum* Sp245 (see Figure 1a) and their assignments ^1^ [19,20,21,22,23,24,25].

Samples of *A. baldaniorum* Sp245		Assignment (Functional Groups)
Without Grinding	With Grinding	
3288	3286	O–H; N–H (amide A in proteins), ν
2962	2961	C–H in –CH3, ν_as_
2930	2929	C–H in >CH2, ν_as_
2878	2877	C–H in –CH3, ν_s_
2854	2854	C–H in >CH2, ν_s_
1737	1729	Ester C=O, ν (PHB; phospholipids)
1654	1653	Amide I (proteins)
1543	1542	Amide II (proteins)
1454	1453	–CH_3_, δ (in proteins, lipids, polyesters, etc.)
1391	1388	COO^−^, ν_s_ (in amino acid side chains and carboxylated polysaccharides) ^2^
1301	1285	C–O–C/C–C–O, ν (in esters; PHB)
1246	1236	C–O–C (esters)/amide III/O–P=O, ν_as_
1188 1124	1187 1127	C–O, C–C, C–OH, ν; C–O–H, C–O–C, δ (polysaccharides)
1084	1085	O–P=O, ν_s_

^1^ Notations: ν—stretching vibrations; ν_s_—symmetric stretching vibrations; ν_as_—antisymmetric stretching vibrations; δ—bending vibrations. ^2^ The corresponding antisymmetric stretching vibrations (ν_as_ of COO^–^, usually of somewhat higher intensities than ν_s_) may vary in wavenumbers (observed commonly around ~1650–1580 cm^−1^) and in microbial biomass are commonly masked by significantly more intensive amide I/II bands of cellular proteins.

**Table 2:** Peak assignments ^1^ in second derivatives of FTIR spectra of biogenic SeNPs obtained using *A. brasilense* Sp7 measured without washing and after 1–3 washing steps (see Figure 6, curves **A**–**D**, respectively) [19,20,21,22,23,24,25,46,47,48].

Functional Groups	SeNPs without Washing	SeNPs after 1 Washing Step	SeNPs after 2 Washing Steps	SeNPs after 3 Washing Steps
C=O (ester), ν	1737 (v.w.) 1728 (v.w.)	1742 1726 (w.)	1737	1734
Amide I (in proteins)	1696 (v.w.) 1679 (v.w.) 1657 1635 (v.w.)	1690 1676 (w.) 1658 (s.) 1644	1690 1675 (w.) 1655 (s.) 1627	1684 1668 (w.) 1654 (s.) 1633
Carboxylate (COO^–^, ν_as_)	1561(v.s.)	1566	1583 (w.)	1556 (w.)
Amide II (in proteins)	1539 (v.w.)	1546 (s.)	1549 (s.)	1542 (s.)
“Tyrosine” band	1518 (w.)	1516	1518	1520
Carboxylate (COO^–^, ν_s_)	1410 (s.)	1409	1413 (w.)	1418 (w.)

^1^ Notations: ν—stretching vibrations; ν_s_—symmetric stretching vibrations; ν_as_—antisymmetric stretching vibrations; δ—bending vibrations; v.s.—very strong, s.—strong, w.—weak, v.w.—very weak.

## Data Availability

The data presented in this study are available in this article.
